# Compressive Strength of Steel Fiber-Reinforced Concrete Employing Supervised Machine Learning Techniques

**DOI:** 10.3390/ma15124209

**Published:** 2022-06-14

**Authors:** Yongjian Li, Qizhi Zhang, Paweł Kamiński, Ahmed Farouk Deifalla, Muhammad Sufian, Artur Dyczko, Nabil Ben Kahla, Miniar Atig

**Affiliations:** 1 James Watt Engineering School, University of Glasgow, Scotland, UK; yongjian4896@outlook.com; 2 School of Architectural Engineering, Huanghuai University, Zhumadian 463000, China; 3 Faculty of Civil Engineering and Resource Management, AGH University of Science and Technology, Mickiewicza 30, 30-059 Kraków, Poland; 4 Structural Engineering and Construction Management Department, Faculty of Engineering and Technology, Future University in Egypt, Cairo 11835, Egypt; ahmed.deifalla@fue.edu.eg; 5 School of Civil Engineering, Southeast University, Nanjing 210096, China; 6 Mineral and Energy Economy Research Institute of the Polish Academy of Sciences, J. Wybickiego 7a, 31-261 Kraków, Poland; arturdyczko@min-pan.krakow.pl; 7 Department of Civil Engineering, College of Engineering, King Khalid University, Abha 61421, Saudi Arabia; nbohlal@kku.edu.sa; 8 Laboratory of Systems and Applied Mechanics, Tunisia Polytechnic School, University of Carthage, La Marsa, Tunis 2078, Tunisia; miniar.atig@gmail.com; 9 Department of Civil Engineering, The Higher National Engineering School of Tunis, University of Tunis, Tunis, Tunisia

**Keywords:** concrete, steel fiber, steel fiber–reinforced concrete, compressive strength, mechanical characteristics, construction materials

## Abstract

Recently, research has centered on developing new approaches, such as supervised machine learning techniques, that can compute the mechanical characteristics of materials without investing much effort, time, or money in experimentation. To predict the 28-day compressive strength of steel fiber–reinforced concrete (SFRC), machine learning techniques, i.e., individual and ensemble models, were considered. For this study, two ensemble approaches (SVR AdaBoost and SVR bagging) and one individual technique (support vector regression (SVR)) were used. Coefficient of determination (R^2^), statistical assessment, and k-fold cross validation were carried out to scrutinize the efficiency of each approach used. In addition, a sensitivity technique was used to assess the influence of parameters on the prediction results. It was discovered that all of the approaches used performed better in terms of forecasting the outcomes. The SVR AdaBoost method was the most precise, with R^2^ = 0.96, as opposed to SVR bagging and support vector regression, which had R^2^ values of 0.87 and 0.81, respectively. Furthermore, based on the lowered error values (MAE = 4.4 MPa, RMSE = 8 MPa), statistical and k-fold cross validation tests verified the optimum performance of SVR AdaBoost. The forecast performance of the SVR bagging models, on the other hand, was equally satisfactory. In order to predict the mechanical characteristics of other construction materials, these ensemble machine learning approaches can be applied.

## 1. Introduction

The bridging effect of discontinuous fibers in fiber-reinforced concrete (FRC) can enhance its strength characteristics. Consequently, incorporation of steel fiber (SF) improves concrete’s compressive strength as well as its toughness and fracture resistance. Concrete’s durability is improved by adding the proper quantity of steel fiber (0–1.5%) [1]. Different types of fibers, i.e., both natural and artificial, are utilized for improving the mechanical characteristics and crack resistance behavior of concrete and cementitious materials [2,3,4,5,6,7,8,9]. Several researchers have carried out studies and presented models for the mechanical characteristics of regular concrete based on a variety of data; however, SFRC contains more factors that require prediction than normal concrete, i.e., the type of fiber, percentage of volume, and the aspect ratio, and creating suitable predictive models is still relatively new. As a consequence, the typical linear and nonlinear regression models struggle to evaluate the compressive strength of SFRC. The difficulty in forecasting the strength of SFRC can be solved by using machine learning approaches [10,11,12,13,14,15,16,17,18,19].

Machine learning techniques are broadly utilized in the computer science and artificial intelligence domains, and their influence on engineering is undeniable. Machine learning techniques have gained attention in civil engineering, mainly for their prediction of the mechanical properties of concrete. These practices can be used to predict outcomes with a high degree of precision. Many studies have been undertaken using machine learning approaches to forecast the strength properties of concrete and its structural elements as well [10,11,12,13,14,15,16,17,18,19,20,21,22,23]. Two machine learning approaches, the individual and ensemble approaches, were employed by Ahmad et al. to forecast concrete compressive strength [24]. Su et al. used different machine learning algorithms to estimate the strength of the link between concrete and fiber-reinforced polymers [25]. Nguyen et al. employed a machine learning approach to assess the compressive characteristics of geo-polymer concrete [26]. Nevertheless, further advances in this field are required to understand and implement these techniques in civil engineering through the use of other machine learning methodologies. The collection of data for the development of models that account for numerous factors such as type of fiber, volume%, and aspect ratio (i.e., the length and diameter of the fiber) is not easy, and there are currently no published studies that define an efficient method for intensity prediction in SFRC. As a result, a machine learning model that predicts the compressive strength of SFRC is developed in this study, and the most appropriate techniques are addressed via a comparison analysis.

There has been a detailed evaluation of the use of ML approaches to predict concrete’s mechanical characteristics [27]. In addition, a number of studies have been conducted to predict the mechanical characteristics of different types of concrete, such as high-performance concrete (HPC) [28], self-healing concrete [29], recycled aggregate concrete (RCA) [30], phase change materials–integrated concrete [31], etc. Steel fiber–reinforced concrete is widely used in civil engineering applications. Therefore, this study considered steel fiber–reinforced concrete (SFRC) with compressive strengths of 26 MPa to 99 MPa. Additionally, the importance of the raw materials was not considered in the datasets on compressive strength in previous studies, and this factor requires evaluation. Therefore, the effects of the input parameters (raw materials) on the compressive strength are studied using sensitivity analysis. However, casting specimens in the lab and curing and evaluating them is a time-consuming and labor-intensive process. Employing innovative approaches such as machine learning techniques to evaluate the mechanical properties of SFRC can address such problems while also reducing experimentation expenses. To estimate the 28-day compressive strength of SFRC, both individual (SVM) and EML (AdaBoost and bagging) approaches were used. The coefficient correlation (R^2^) value was used to validate the quality of each model. To compare the outcomes of each method, the statistical error, namely the mean-absolute error (MSE), root-mean-square error (RMSE), and k-fold cross validation tests were performed. Sensitivity analysis was used to examine the contribution of the model parameters to the outcome predictions. Scholars working in the civil engineering field could benefit from this study since it allows them to predict strength attributes without having to spend time in the lab.

## 2. Data Description

The information was gathered from 17 publications [32,33,34,35,36,37,38,39,40,41,42,43,44,45,46,47,48] as shown in Table S1. Since the goal of this study is to develop a basic machine learning model for SFRC and forecast its mechanical parameters, this dataset was constructed entirely using data from hook-end steel fiber–concrete. Despite the fact that the dataset had a large number of variables, only those that were shown to be fundamentally influenced were chosen and preprocessed. As a result, the dataset has a total of t10 features, including input data and output data. The following 10 factors were considered for the forecasting of SFRC compressive strength, since they are the most fundamental mechanical characteristics of SFRC, and each of these variables impact the compressive strength of SFRC.

*Water and Cement:* The ratio of water to cement (W/C) has a big impact on concrete strength. According to Abbass et al., when the W/C ratio rises, the compressive strength drops [44]. Similarly, Reddy et al. performed tests on self-consolidating concrete based on the W/C ratio and concluded that it had a significant impact [49]. Nili et al. found that the water–cement ratio was a factor influencing SFRC compressive strength, and it was chosen as a variable [50].

*Sand and Aggregate:* The impact of the sand and aggregate ratio (s/a) on the strength properties of SFRC has been identified as a critical element. Kim et al. observed that increasing the s/a ratio caused an increase in SFRC’s compressive strength [51]. Chitlange et al. found a significant variation in compressive strength based on the s/a ratio [52]. As a result, the sand and aggregate content was chosen as a key variable in the development of the ML models.

*Superplasticizer:* A superplasticizer is an additive commonly used in the manufacture of high-strength concrete. Khan et al. reported that the combination of superplasticizer and pozzolanic ingredients can enhance concrete’s mechanical qualities [53]. Furthermore, a superplasticizer is essential because concrete requires a larger water content, which causes bleeding. Aruntas et al. found that when the superplasticizer concentration rises to 1.5%, the workability and compressive strength increase [54]. As a result, superplasticizer was chosen as a feature in the ML models to quantify its direct effect on SFRC’s compressive strength.

*Silica fume:* Many earlier researchers have shown the significant impact of silica fume on concrete strength. According to Köksal et al., increasing the silica fume concentration improves compressive strength [42]. Nili et al. found an enhancement in concrete compressive strength with increased content of silica fume [55]. As a result, the concentration of silica fume was discovered as an impact factor on the strength properties of SFRC and was chosen as a characteristic.

*Fly ash:* It is a popular ingredient that can increase concrete performance in both states i.e., fresh (workability) and hardened (strength), over time. According to R. Saravana and A. Sumathi, incorporating fly ash into SFRC enhanced the compressive strength over time [56]. The impact of fly ash and steel fibers on the strength qualities of pozzolana cement concrete was explored by Muntadher, A.C. and Srivastava, V. [57]. Ashish, K.S. inspected the effect of fly ash on concrete durability and found that the concrete’s compressive strength was lower than predicted at first but steadily increased with time [58]. Using a machine learning method, Mohammad, M.R. et al. evaluated the mechanical characteristics of fly ash concrete [59]. Fly ash was chosen as a criterion because of its importance in concrete characteristics.

*Steel Fiber volume, length, and diameter:* In prior investigations it was found that steel fiber volume, length, and diameter had a significant influence on the concrete’s compressive strength. Yazc et al. found that the fiber volume fraction increases compressive strength with lower aspect ratios, while there was no significant development found in compressive strength with higher aspect ratios [1]. Furthermore, the experimental investigation of Köksal et al. revealed that the strength properties of SFRC improve as a consequence of utilizing fiber volume fractions of up to 1% [42]. As a result, fiber volume, length, and diameter must be considered as variables in the ML models.

**Figure 1:**
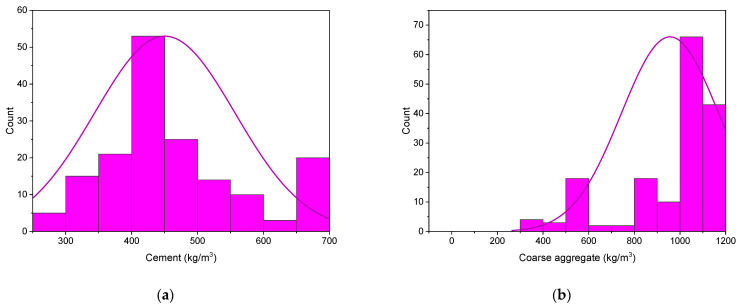

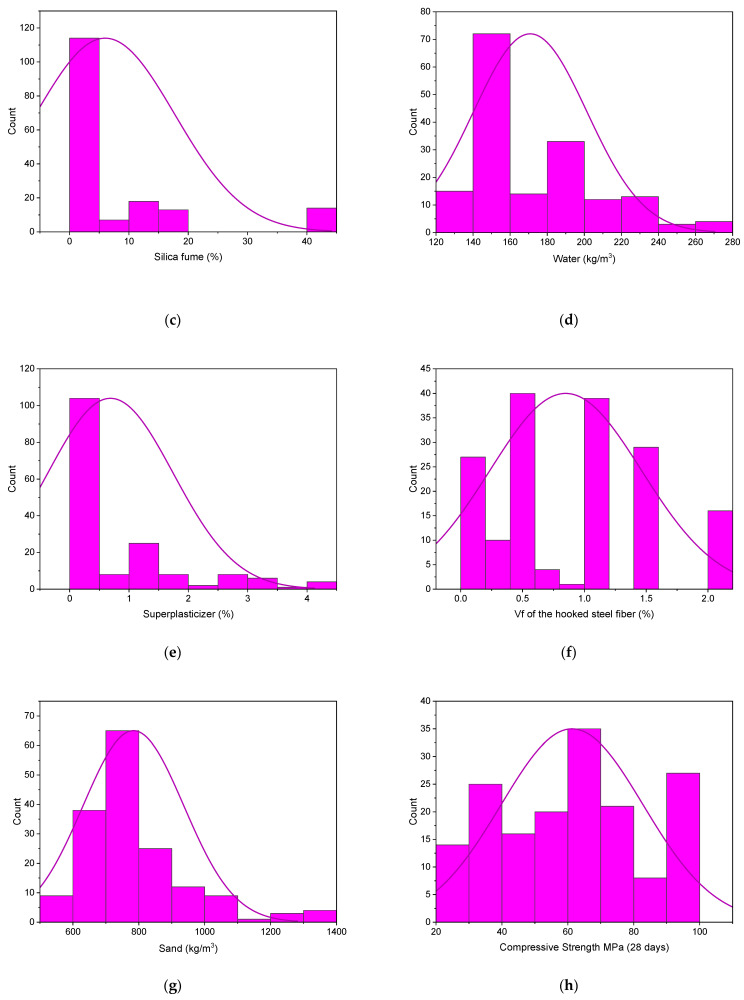
Relative frequency patterns of input and output factors: (**a**) cement; (**b**) coarse aggregate; (**c**) silica fume; (**d**) water; (**e**) superplasticizer; (**f**) volume fraction of steel hooked fibers; (**g**) sand; and (**h**) compressive strength.

To obtain the intended result, ML approaches require a range of input variables [60]. The information utilized to estimate SFRC’s compressive strength is obtained from the literature. Only the findings for 28-day compressive strength were separated from the data collected for further study. The models contained cement, water, sand, coarse aggregate, superplasticizer, silica fume, fly ash, steel fiber, fiber length, and fiber diameter as inputs, with just one variable, i.e., compressive strength, as a resultant output. This study used 166 data points for the 28-day compressive strength prediction of SFRC (mix proportions). The database includes normal strength and high-strength SFRC with compressive strengths ranging from 26 MPa to 99 MPa. Table 1 displays the statistical analysis outcomes of the input variables, i.e., mean, standard error, median, mode, standard deviation, range, minimum, and maximum values. Furthermore, the relative frequency pattern distribution of all input parameters is depicted in Figure 1.

## 3. Research Strategy

**Figure 2:**
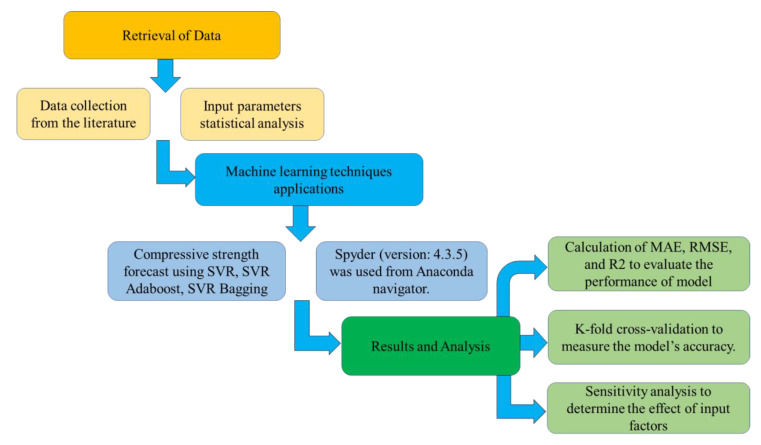
Research methodology in the current study.

The ML models were run with Python code using Anaconda software. The Anaconda navigator is a desktop graphical user interface featured in the Anaconda program that allows for the running of apps that provide guidance through the Conda packages, channels, and environments without having to use command-line techniques. It is also a source for Python and R programming languages for data science and machine learning applications, with an emphasis on package development and maintenance. This work used three techniques to estimate the compressive strength of SFRC, i.e., SVR, AdaBoost, and bagging. Spyder (version: 4.3.5) was chosen from the Anaconda navigator for model execution. The degree of accuracy was represented by the R^2^ value of the predicted result from all models. R^2^ values typically range from 0 to 1, with a higher number suggesting greater accuracy between the measured and predicted outcomes. Furthermore, statistical checks, error evaluation (including MAE, RMSE), and k-fold cross-validation were done to assess the performance of the models employed in this study. A sensitivity analysis was also performed to identify the influence of all input variables. Figure 2 illustrates the research strategy as a flowchart.

## 4. Results and Discussions

### 4.1. Statistical Analysis Explanation

**Figure 3:**
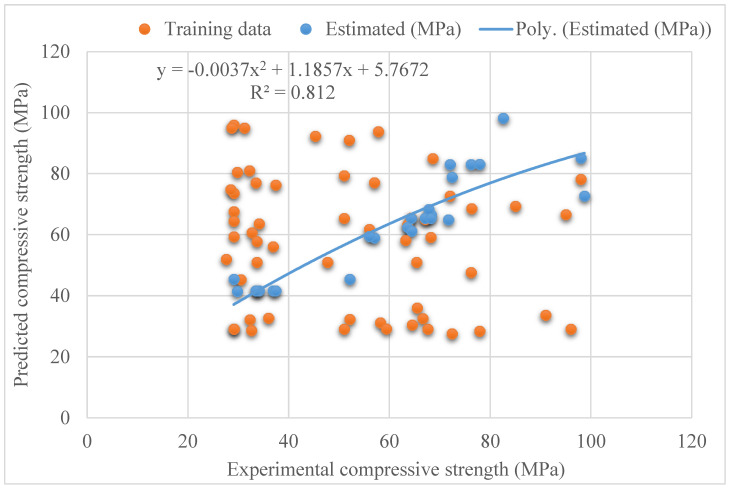
Relationship for support vector regression model: experimental and estimated results.

**Figure 4:**
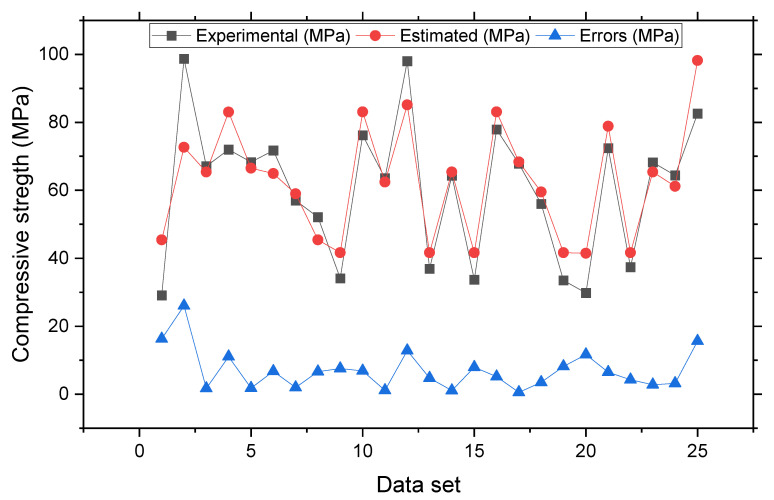
Experimental and estimated values and error distribution for support vector regression model.

Figure 3 depicts the pattern of statistical analysis for the actual and predicted outcomes of SFRC compressive strength after 28 days utilizing the SVR model. The SVR delivers results that are within the permitted range and have a minimal difference between real and expected results. The R^2^ = 0.81 demonstrates that the model performs well in terms of calculating outcomes. Figure 4 shows the distribution of investigational and estimated outcomes, and the SVR model’s errors. In the dispersal, the largest, lowermost, and average values of error were 26.02, 0.53, and 7.03 MPa, respectively. It was discovered that 44% of the erroneous values were less than 5 MPa, 28% were between 5 and 10 MPa, and 28% were higher than 10 MPa. These statistics represent the level of agreement between the projected and actual results.

**Figure 5:**
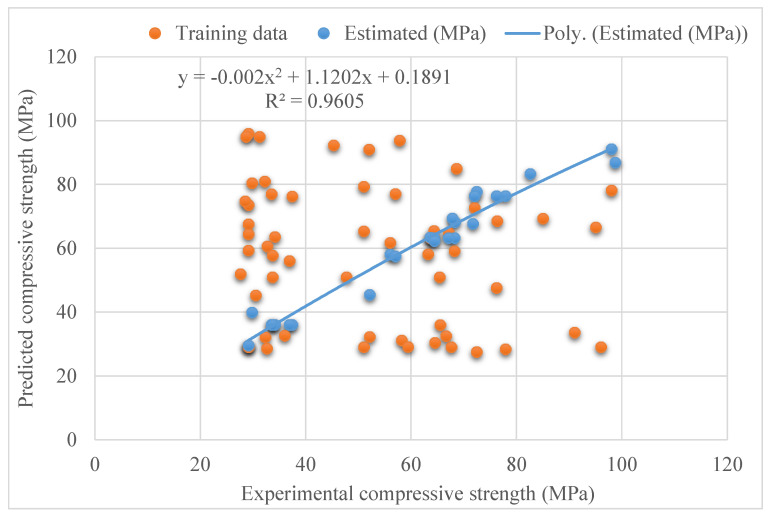
Connection between experimental and estimated results for SVR AdaBoost model.

**Figure 6:**
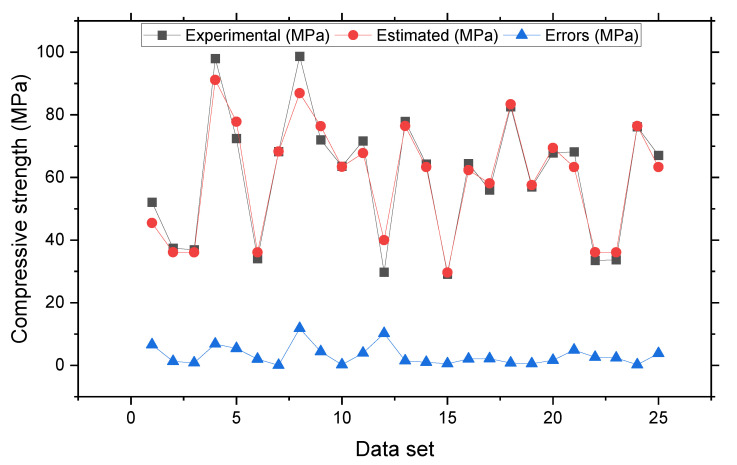
Experimental and estimated values and error distribution for SVR AdaBoost model.

Figure 5 and Figure 6 depict the AdaBoost model’s outputs. Figure 5 displays the connection between the genuine and predicted outcomes, with R^2^ = 0.96, which is greater than that of SVR model, implying that the AdaBoost approach performs better than SVR. Figure 6 depicts the AdaBoost model’s distribution of actual and projected values as well as errors. The distribution’s highest, lowest and average values of the errors were 11.80, 0.005, and 3.10 MPa, respectively. According to the findings, 80% of the erroneous readings were less than 5 MPa, 12% were in the range of 5–10 MPa, and 8% were more than 10 MPa. The AdaBoost model can more effectively predict SFRC’s compressive strength based on the R^2^ and error distribution of the SVM and AdaBoost models.

**Figure 7:**
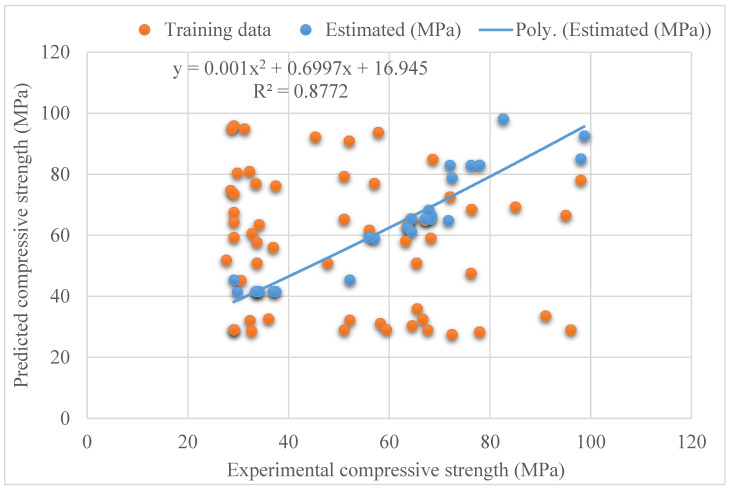
Experimental and estimated outcome relationship for SVR bagging model.

**Figure 8:**
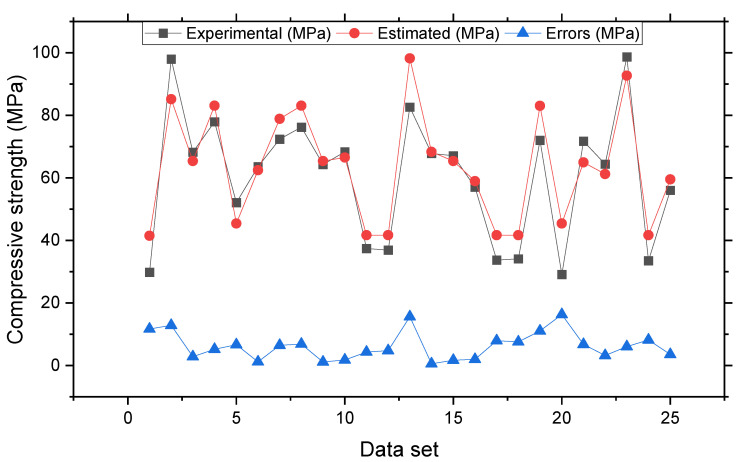
Experimental and estimated values and error distribution for SVR bagging model.

The connection of the actual and expected outcomes for the model of SVR bagging is shown in Figure 7. The SVR bagging model has R^2^ = 0.87, showing that this model is more precise than the SVR but less precise than SVR AdaBoost models. Figure 8 also shows the SVR bagging distribution for the actual and projected values and errors. The values 16.3, 0.53, and 6.5 MPa were the greatest, lowest, and average errors, respectively. It was discovered that 52% of the erroneous values were less than 5 MPa, 28% were between 5 Mpa and 10 MPa, and 20% were larger than 10 MPa. This investigation demonstrated that the SVR AdaBoost model had superior precision compared with the SVR and SVR AdaBoost models due to its lower error and higher R^2^ readings. In addition, the ML techniques, i.e., SVR AdaBoost and SVR bagging, used the sub-models to obtain the best assessments that yielded nearly perfect outcomes. Wang et al. [61] reported that AdaBoost machine learning approaches better predict the compressive strength of geopolymer composites. Zhu et al. [62] used machine learning to forecast the splitting tensile strength (STS) of concrete containing recycled aggregate (RA) and revealed that the precision level of the bagging model was better. Ahmad et al. [63] studied boosting, and the AdaBoost ML approaches to predict the compressive strength of high-calcium fly-ash-based geopolymer. The AdaBoost and bagging models yielded better results. As a result, this study found that the ML methods (SVR AdaBoost and SVR bagging) were more accurate than the individual strategy in predicting outcomes (SVR).

### 4.2. Cross-Validation Using the K-Fold Scale

To assess the legitimacy of the model during execution, the k-fold cross-validation technique was utilized. This technique is regularly employed to check the correctness of the model in which the data set spread and split into 10 groups [60,61,62,63,64,65]. One group is used to verify the model, while the other 9 are utilized for training; 70% of the data set was utilized in the training process of the models, while the remaining 30% of the data were used for testing and validation of the models. If the R^2^ value is high and the errors, i.e., the MAE and RMSE values, are low, then the model is considered more accurate. To achieve a decent outcome, the technique must be performed 10 times. The model’s high accuracy is due to this complete methodology. In addition, as shown in Table 2, all models were subjected to statistical analysis of errors, namely MSE and RMSE. The response of the models to the estimate was evaluated by statistical analysis, employing Equations (1) and (2), taken from the literature [66]:

(1) $$\mathrm{MAE}=\frac{1}{n}\overset{n}{\underset{i=1}{\sum }}\left|x_{i}-x\right|$$

(2) $$\mathrm{RMSE}=\sqrt{\sum \frac{{\left(y_{pred}-y_{ref}\right)}^{2}}{n}}$$

where n = the total number of sampled data, x, $y_{ref}$ = reference values of the data sample, and $x_{i}$, $y_{pred}$ = model-predicted values.

**Figure 9:**
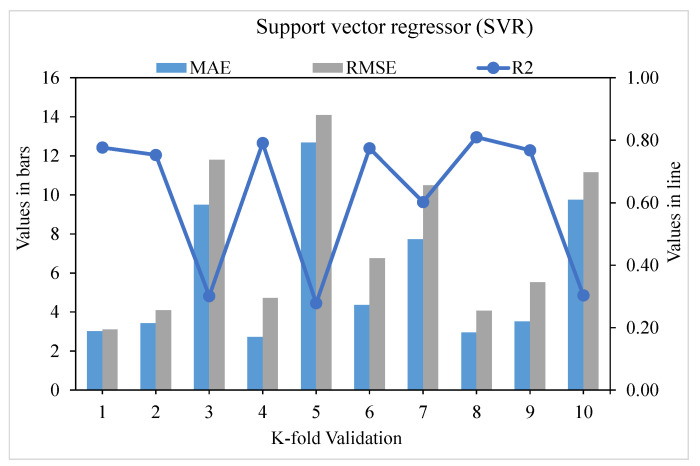
Support vector regression model with K-fold cross-validation representation.

**Figure 10:**
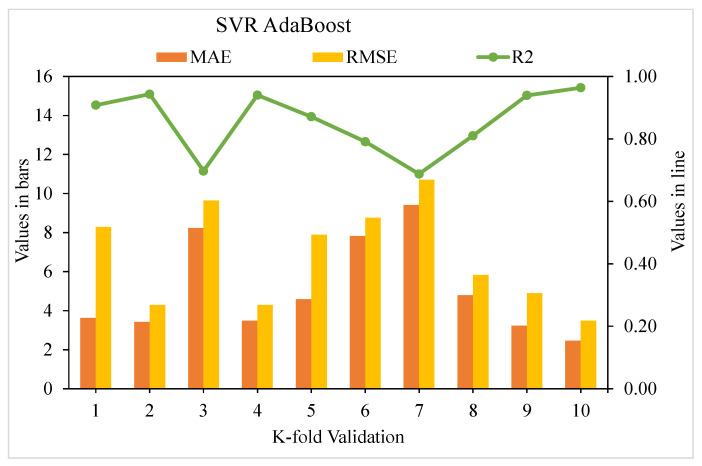
K-fold cross-validation representation for the AdaBoost model.

**Figure 11:**
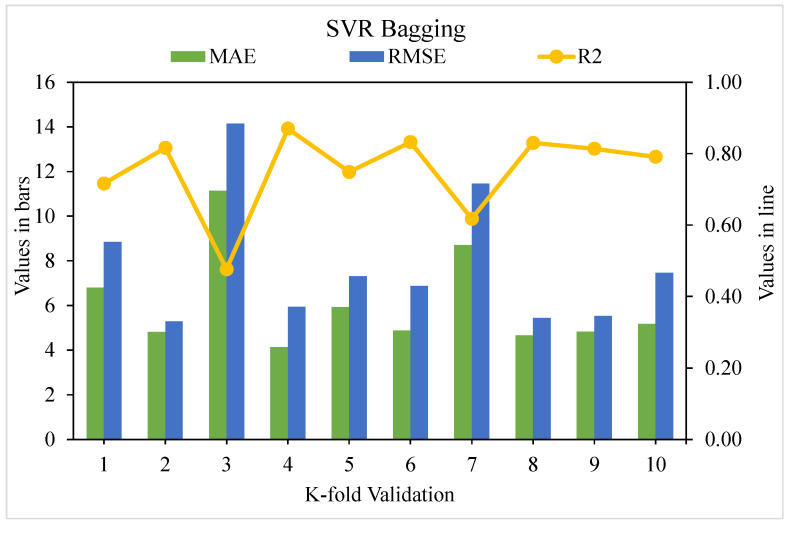
K-fold cross-validation representation for bagging model.

Figure 9, Figure 10 and Figure 11 show the distributions of MAE, RMSE, and R^2^ for the k-fold cross-validation of models SVR, SVR AdaBoost, and SVR bagging, respectively. As shown in Figure 9, the SVR model’s best, lowest, and average R^2^ values are 0.80, 0.55, and 0.69, respectively. The greatest, lowermost, and average R^2^ values for the AdaBoost model were 0.90, 0.60, and 0.76, respectively, as illustrated in Figure 10. The greatest, least, and R^2^ average values for the bagging model were 0.92, 0.61, and 0.79, respectively, as presented in Figure 11. When the error values for the SVR model were compared, the average MAE and RMSE were 9.04 and 13.62, respectively. The AdaBoost model had average MAE and RMSE values of 8.18 and 11.63, respectively, whereas the bagging model had average MAE and RMSE values of 6.51 and 8.39, respectively. The AdaBoost model with the lowermost error and the highest R^2^ value performed the best in terms of predicting outcomes. Table 3 shows the k-fold analysis findings for the utilized models, including the MAE, RMSE, and R^2^ values.

### 4.3. Sensitivity Analysis

**Figure 12:**
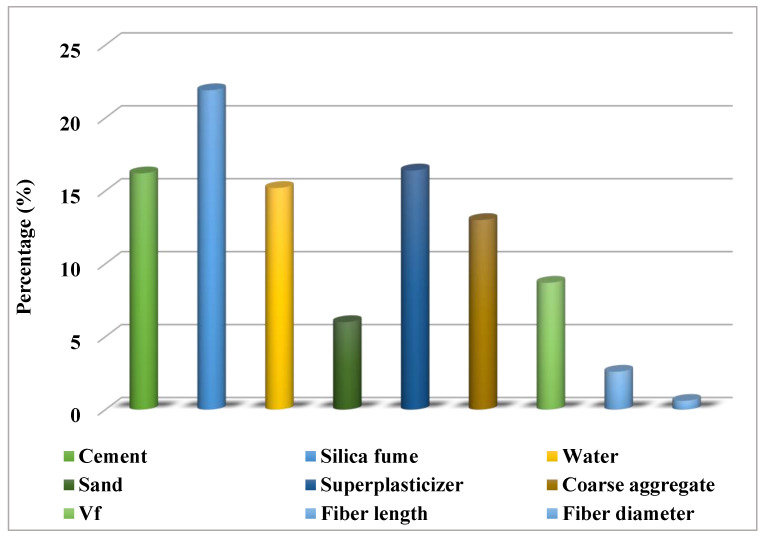
The input variable contribution to the forecast. Cement, water, superplasticizer, silica fume, coarse aggregate, sand, Vf (volume factor), fiber length, and fiber diameter.

The goal of this study was to determine how input factors affect the forecasting of SFRC compressive strength. The input factors showed a significant impact on the predicted result [67]. Figure 12 depicts the impact of input variables on the prediction of SFRC’s compressive strength. The investigation found that silica fume was the utmost essential element, accounting for 21.9% of the total, along with cement (16.2%) and super plasticizer (16.4%). The other input factors had a smaller impact on the prediction of the compressive strength of SFRC, with coarse aggregate accounting for 13%, water 15.2%, and sand 6%. The number of input factors and data points utilized in the design of the models were proportional to the results of the sensitivity analysis. The influence of input variables on the model output was checked using Equations (3) and (4). As can be seen in Figure 12, cement, silica fume, water and superplasticizer had the greatest effect on the compressive strength. This is obvious as these are the main factors contributing to the strength development of SFRC, with cement and silica fume especially increasing the compressive strength due to their hydration and pozzolanic activity. Similarly, water and superplasticizer are also main factors as a decreased *w/c* ratio results in increased compressive strength. Similarly, coarse aggregate, steel fiber volume, and length also contribute to the strength, as explained in Section 2.

(3) $$N_{i}=f_{max}\left(x_{i}\right)-f_{min}\left(x_{i}\right)$$

(4) $$S_{i}=\frac{N_{i}}{\sum_{j-i}^{n}N_{j}}$$

The highest and lowest projected outputs over the $i^{th}$ output are represented by $f_{max}\left(x_{i}\right)$ and $f_{min}\left(x_{i}\right)$, respectively.

## 5. Discussion

**Figure 13:**
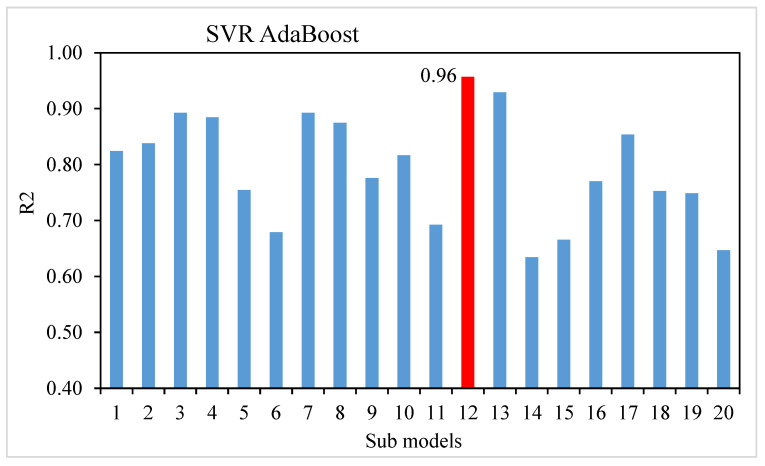
The coefficient of correlation (R^2^) values of the SVR AdaBoost sub-model.

**Figure 14:**
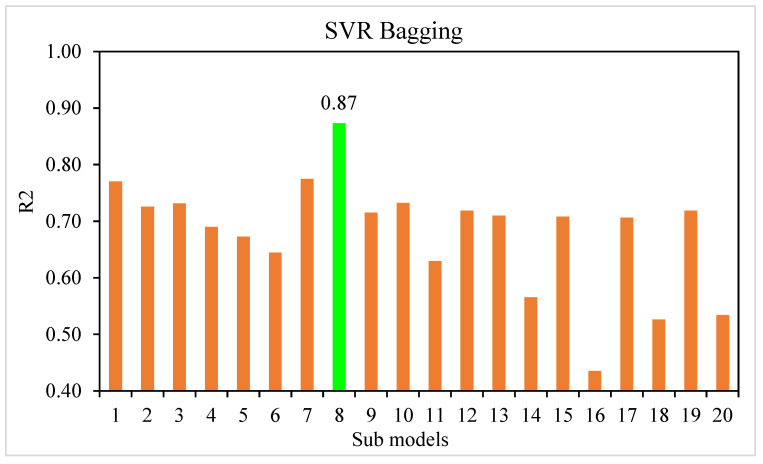
SVR bagging sub-model’s coefficient of correlation (R^2^) values.

This research work aimed to determine how ML methods can be utilized to predict the compressive strength of SFRC. Learning rates and other features that specifically affect ensemble approaches can be used as tuning parameters for the model used in the ensemble techniques. Boosting ensemble models (20 each) with 10, 20, 30, …, 200 component sub-models were developed for base learners in this work, and correlation with high coefficient values was utilized to identify the best model. The randomization technique further revealed the statistical importance. The test was carried out by (i) permuting the data set’s activity values repeatedly, (ii) generating models from the permuted values, and (iii) comparing the resulting scores to the score of the original model derived from non-randomized activity values. If the original model is statistically significant, the score from the permuted data should be significantly higher. Three machine learning approaches were considered: one individual, i.e., SVR, and two ensemble, i.e., SVR AdaBoost and SVR bagging. The prediction performance of each approach was evaluated to see which approach is the most precise in prediction. When compared, the SVR and SVR bagging models yielded R^2^ values of 0.81 and 0.87, respectively. The SVR AdaBoost outcome was more exact, with an R^2^ value of 0.96. To confirm the efficiency of all models, statistical analysis and the k-fold cross-validation technique were applied. The models work better with minimal error levels. The ML techniques frequently take advantage of the vulnerable intern by developing sub-models trained on data and maximized to enhance the value of R^2^. Figure 13 and Figure 14 display the range of R^2^ values for the sub-models, i.e., the SVR AdaBoost and SVR bagging approaches. The peak, lowermost, and average R^2^ values of the AdaBoost sub-models were 0.96, 0.63, and 0.79, respectively. For the bagging sub-models, the largest, least, and average R^2^ values were 0.87, 0.44, and 0.68, respectively. These outcomes indicate that SVR AdaBoost is more accurate than the bagging sub-models. Sensitivity analysis was also carried out to determine how each input parameter affected the SFRC’s predicted compressive strength. The sensitivity analysis looked at how much each of the 10 input factors influenced the expected outcome.

## 6. Conclusions

This research aimed to practice individual and ensembled ML approaches to estimate the 28-day compressive strength of SFRC. To predict the outcomes, the researchers used support vector regression (SVR), AdaBoost, and bagging models. The following are the findings of this investigation:

- Individual approaches were less accurate than EML procedures in forecasting SFRC’s compressive strength, while the SVR bagging model displayed the highest accuracy.
- The SVR bagging model outperformed the SVR AdaBoost ensembled machine learning technique in the forecasting of the 28-day compressive strength of SFRC.
- The SVR, SVR AdaBoost, and SVR bagging models have coefficient of determination (R2) values of 0.81, 0.96, and 0.87, respectively. All of the models’ outputs are within acceptable bounds, with little variance from the exact results.
- The models’ performances were demonstrated by the k-fold cross-validation test and statistical analysis, which revealed that the SVR bagging model outperformed the other models investigated in terms of prediction.
- To determine how much the input parameters mattered, a sensitivity analysis was utilized, and it was discovered that cement, water, silica fume, sand, superplasticizer, coarse aggregate, Vf, fiber length, and fiber diameter contributed 16.2%, 15.2%, 21.9%, 6%, 16.4%, 13%, 8.7%, 2.6%, and 0.6%, respectively, to the outcome predictions.
- The unique ensemble machine learning algorithms, especially that of the SVR bagging model, can effectively estimate concrete strength qualities without the requirement for prolonged casting and testing process.

However, other prediction approaches [68,69,70,71,72], such as density functional theory can be used to understand atomistic details of crack and structural failure (which can be fed directly into machine learning approaches instead of just obtaining data from the literature). These approaches can reveal mechanisms for the strength of structures in a more unbiased way, which should be studied in future. In addition, this research was also limited to the prediction of compressive strength at 28 days with 10 input parameters and did not consider specimen size and the curing age of concrete. Indeed, proper database and testing must be applied as these are vital elements for engineering applications. This study was based on a wide range of data sets with nine input variables; however, the database and more input parameters, such as specimen size and curing age, among others, need to be generated in future for a better response from the employed models.

## Figures and Tables

**Table 1:** Statistical analysis of input and output variables.

	Cement (kg/m^3^)	Water (kg/m^3^)	Sand (kg/m^3^)	Coarse Aggregate (kg/m^3^)	Superplasticizer (%)	Silica Fume %	Fly Ash %	Steel Fiber (%)	Fiber Length (mm)	Fiber Dia (mm)	Compressive Strength MPa (28 Days)
**Mean**	445.8	170.8	783.7	940.7	0.9	6.0	1.4	0.8	40.5	0.6	61.3
**Standard Error**	8.2	2.4	11.9	19.9	0.1	0.9	0.4	0.0	1.2	0.0	1.7
**Median**	400.0	157.8	743.0	1050.5	0.2	0.0	0.0	1.0	35.0	0.6	62.8
**Mode**	400.0	152.0	835.0	1047.0	0.0	0.0	0.0	0.5	60.0	0.8	29.1
**Standard Deviation**	105.4	30.7	153.3	256.8	1.8	11.7	5.7	0.6	16.1	0.2	21.6
**Range**	400.0	137.0	768.0	1170.0	9.0	43.0	30.0	2.0	60.0	0.9	73.1
**Minimum**	280.0	133.0	582.0	0.0	0.0	0.0	0.0	0.0	0.0	0.0	26.1
**Maximum**	680.0	270.0	1350.0	1170.0	9.0	43.0	30.0	2.0	60.0	0.9	99.2
**Count**	166.0	166.0	166.0	166.0	166.0	166.0	166.0	166.0	166.0	166.0	166.0

**Table 2:** Statistical analysis of the approaches used.

Models	MAE (MPa)	RMSE (MPa)	R^2^
Support vector regression	7.0	9.1	0.81
SVR AdaBoost	4.4	8.0	0.96
SVR bagging	6.2	7.6	0.87

**Table 3:** K-fold cross-validation results.

K-Fold	SVR			SVR AdaBoost			SVR Bagging		
	MAE	RMSE	R^2^	MAE	RMSE	R^2^	MAE	RMSE	R^2^
1	3.02	3.11	0.78	3.62	8.29	0.91	6.79	8.84	0.72
2	3.43	4.10	0.75	3.43	4.29	0.94	4.81	5.28	0.82
3	9.49	11.80	0.30	8.24	9.65	0.70	11.15	14.15	0.48
4	2.72	4.73	0.79	3.49	4.29	0.94	4.14	5.93	0.87
5	12.69	14.10	0.28	4.58	7.89	0.87	5.93	7.31	0.75
6	4.36	6.76	0.77	7.82	8.76	0.79	4.88	6.88	0.83
7	7.73	10.50	0.60	9.40	10.70	0.69	8.72	11.46	0.62
8	2.95	4.07	0.81	4.79	5.83	0.81	4.66	5.45	0.83
9	3.52	5.52	0.77	3.23	4.90	0.94	4.83	5.53	0.81
10	9.76	11.17	0.30	2.46	3.49	0.96	5.16	7.46	0.79

## Data Availability

Data is already available in the supplemtray file.

## Supplementary Materials

The following supporting information can be downloaded at: https://www.mdpi.com/article/10.3390/ma15124209/s1, Table S1: Dataset used for prediction.

## References

[1] Nili M., Azarioon A., Danesh A., Deihimi A. (2018). Experimental study and modeling of fiber volume effects on frost resistance of fiber reinforced concrete. Int. J. Civ. Eng..

[2] Khan M., Ali M. (2019). Improvement in concrete behavior with fly ash, silica-fume and coconut fibres. Constr. Build. Mater..

[3] Li L., Khan M., Bai C., Shi K. (2021). Uniaxial tensile behavior, flexural properties, empirical calculation and microstructure of multi-scale fiber reinforced cement-based material at elevated temperature. Materials.

[4] Cao M., Mao Y., Khan M., Si W., Shen S. (2018). Different testing methods for assessing the synthetic fiber distribution in cement-based composites. Constr. Build. Mater..

[5] Khan M., Cao M., Xie C., Ali M. (2022). Hybrid fiber concrete with different basalt fiber length and content. Struct. Concr..

[6] Khan M., Cao M., Hussain A., Chu S.H. (2021). Effect of silica-fume content on performance of CaCO_3_ whisker and basalt fiber at matrix interface in cement-based composites. Constr. Build. Mater..

[7] Arshad S., Sharif M.B., Irfan-ul-Hassan M., Khan M., Zhang J.L. (2020). Efficiency of Supplementary Cementitious Materials and Natural Fiber on Mechanical Performance of Concrete. Arab. J. Sci. Eng..

[8] Xie C., Cao M., Guan J., Liu Z., Khan M. (2021). Improvement of boundary effect model in multi-scale hybrid fibers reinforced cementitious composite and prediction of its structural failure behavior. Compos. Part B Eng..

[9] Thomas B.S., Yang J., Bahurudeen A., Abdalla J.A., Hawileh R.A., Hamada H.M., Nazar S., Jittin V., Ashish D.K. (2021). Sugarcane bagasse ash as supplementary cementitious material in concrete—A review. Mater. Today Sustain..

[10] Young B.A., Hall A., Pilon L., Gupta P., Sant G. (2019). Can the compressive strength of concrete be estimated from knowledge of the mixture proportions?: New insights from statistical analysis and machine learning methods. Cem. Concr. Res..

[11] Akande K.O., Owolabi T.O., Twaha S., Olatunji S.O. (2014). Performance Comparison of SVM and ANN in Predicting Compressive Strength of Concrete. IOSR J. Comput. Eng..

[12] Chou J.S., Tsai C.F., Pham A.D., Lu Y.H. (2014). Machine learning in concrete strength simulations: Multi-nation data analytics. Constr. Build. Mater..

[13] Duan J., Asteris P.G., Nguyen H., Bui X.N., Moayedi H. (2021). A novel artificial intelligence technique to predict compressive strength of recycled aggregate concrete using ICA-XGBoost model. Eng. Comput..

[14] Gupta S.M. (2007). Support Vector Machines based Modelling of Concrete Strength. World Acad. Sci. Eng. Technol..

[15] Chou J.S., Pham A.D. (2013). Enhanced artificial intelligence for ensemble approach to predicting high performance concrete compressive strength. Constr. Build. Mater..

[16] Deepa C., SathiyaKumari K., Sudha V.P. (2010). Prediction of the Compressive Strength of High Performance Concrete Mix using Tree Based Modeling. Int. J. Comput. Appl..

[17] Erdal H.I. (2013). Two-level and hybrid ensembles of decision trees for high performance concrete compressive strength prediction. Eng. Appl. Artif. Intell..

[18] Nafees A., Khan S., Javed M.F., Alrowais R., Mohamed A.M., Mohamed A., Vatin N.I. (2022). Forecasting the Mechanical Properties of Plastic Concrete Employing Experimental Data Using Machine Learning Algorithms: DT, MLPNN, SVM, and RF. Polymers.

[19] Nafees A., Javed M.F., Khan S., Nazir K., Farooq F., Aslam F., Musarat M.A., Vatin N.I. (2021). Predictive modeling of mechanical properties of silica fume-based green concrete using artificial intelligence approaches: MLPNN, ANFIS, and GEP. Materials.

[20] Khan M.A., Aslam F., Javed M.F., Alabduljabbar H., Deifalla A.F. (2022). New prediction models for the compressive strength and dry-thermal conductivity of bio-composites using novel machine learning algorithms. J. Clean. Prod..

[21] Salem N.M., Deifalla A. (2022). Evaluation of the Strength of Slab-Column Connections with FRPs Using Machine Learning Algorithms. Polymers.

[22] Ebid A., Deifalla A. (2022). Using Artificial Intelligence Techniques to Predict Punching Shear Capacity of Lightweight Concrete Slabs. Materials.

[23] Dong W., Huang Y., Lehane B., Ma G. (2020). XGBoost algorithm-based prediction of concrete electrical resistivity for structural health monitoring. Autom. Constr..

[24] Ahmad A., Farooq F., Niewiadomski P., Ostrowski K., Akbar A., Aslam F., Alyousef R. (2021). Prediction of compressive strength of fly ash based concrete using individual and ensemble algorithm. Materials.

[25] Su M., Zhong Q., Peng H., Li S. (2021). Selected machine learning approaches for predicting the interfacial bond strength between FRPs and concrete. Constr. Build. Mater..

[26] Nguyen K.T., Nguyen Q.D., Le T.A., Shin J., Lee K. (2020). Analyzing the compressive strength of green fly ash based geopolymer concrete using experiment and machine learning approaches. Constr. Build. Mater..

[27] Ben Chaabene W., Flah M., Nehdi M.L. (2020). Machine learning prediction of mechanical properties of concrete: Critical review. Constr. Build. Mater..

[28] Castelli M., Vanneschi L., Silva S. (2013). Prediction of high performance concrete strength using Genetic Programming with geometric semantic genetic operators. Expert Syst. Appl..

[29] Suleiman A.R., Nehdi M.L. (2017). Modeling self-healing of concrete using hybrid genetic algorithm-artificial neural network. Materials.

[30] Zhang J., Huang Y., Aslani F., Ma G., Nener B. (2020). A hybrid intelligent system for designing optimal proportions of recycled aggregate concrete. J. Clean. Prod..

[31] Marani A., Nehdi M.L. (2020). Machine learning prediction of compressive strength for phase change materials integrated cementitious composites. Constr. Build. Mater..

[32] Soulioti D.V., Barkoula N.M., Paipetis A., Matikas T.E. (2011). Effects of fibre geometry and volume fraction on the flexural behaviour of steel-fibre reinforced concrete. Strain.

[33] Yoo D.Y., Yoon Y.S., Banthia N. (2015). Flexural response of steel-fiber-reinforced concrete beams: Effects of strength, fiber content, and strain-rate. Cem. Concr. Compos..

[34] Jang S.J., Yun H. (2018). Do Combined effects of steel fiber and coarse aggregate size on the compressive and flexural toughness of high-strength concrete. Compos. Struct..

[35] Johnson D.A., Pedersen N., Jacobsen C.B. (2014). Effect of steel fibers on flexural behaviour of normal and high strength concrete. Int. J. Civ. Environ. Eng..

[36] Dinh N.H., Park S.H., Choi K.K. (2021). Effect of dispersed micro-fibers on tensile behavior of uncoated carbon textile-reinforced cementitious mortar after high-temperature exposure. Cem. Concr. Compos..

[37] Thomas J., Ramaswamy A. (2007). Mechanical Properties of Steel Fiber-Reinforced Concrete. J. Mater. Civ. Eng..

[38] Sivakumar A., Santhanam M. (2007). Mechanical properties of high strength concrete reinforced with metallic and non-metallic fibres. Cem. Concr. Compos..

[39] Afroughsabet V., Ozbakkaloglu T. (2015). Mechanical and durability properties of high-strength concrete containing steel and polypropylene fibers. Constr. Build. Mater..

[40] Atiş C.D., Karahan O. (2009). Properties of steel fiber reinforced fly ash concrete. Constr. Build. Mater..

[41] Lee J.H., Cho B., Choi E. (2017). Flexural capacity of fiber reinforced concrete with a consideration of concrete strength and fiber content. Constr. Build. Mater..

[42] Köksal F., Altun F., Yiǧit I., Şahin Y. (2008). Combined effect of silica fume and steel fiber on the mechanical properties of high strength concretes. Constr. Build. Mater..

[43] Yoon E.S., Park S.B. (2006). An experimental study on the mechanical properties and long-term deformations of high-strength steel fiber reinforced concrete. J. Korean Soc. Civ. Eng..

[44] Abbass W., Khan M.I., Mourad S. (2018). Evaluation of mechanical properties of steel fiber reinforced concrete with different strengths of concrete. Constr. Build. Mater..

[45] Yoo D.Y., Yoon Y.S., Banthia N. (2015). Predicting the post-cracking behavior of normal- and high-strength steel-fiber-reinforced concrete beams. Constr. Build. Mater..

[46] Lee H.-H., Lee H.-J. (2004). Characteristic Strength and Deformation of SFRC Considering Steel Fiber Factor and Volume fraction. J. Korea Concr. Inst..

[47] Oh Y.H. (2008). Evaluation of Flexural Strength for Normal and High Strength Concrete with Hooked Steel Fibers. J. Korea Concr. Inst..

[48] Song P., Hwang S. (2004). Mechanical properties of high-strength steel fiber-reinforced concrete. Constr. Build. Mater..

[49] Reddy V.M., Rao D.M.V.S. (2014). Effect of w/c ratio on workability and mechanical properties of high strength Self Compacting Concrete (M70 grade). IOSR J. Mech. Civ. Eng..

[50] Nili M., Afroughsabet V. (2010). Combined effect of silica fume and steel fibers on the impact resistance and mechanical properties of concrete. Int. J. Impact Eng..

[51] Kim J.J., Kim D.J., Kang S.T., Lee J.H. (2012). Influence of sand to coarse aggregate ratio on the interfacial bond strength of steel fibers in concrete for nuclear power plant. Nucl. Eng. Des..

[52] Chitlange M.R., Pajgade P.S. (2010). Strength appraisal of artificial sand as fine aggregate in SFRC. J. Eng. Appl. Sci..

[53] Khan M., Ali M. (2018). Effect of super plasticizer on the properties of medium strength concrete prepared with coconut fiber. Constr. Build. Mater..

[54] Aruntaş H.Y., Cemalgil S., Şimşek O., Durmuş G., Erdal M. (2008). Effects of super plasticizer and curing conditions on properties of concrete with and without fiber. Mater. Lett..

[55] Nili M., Afroughsabet V. (2012). Property assessment of steel-fibre reinforced concrete made with silica fume. Constr. Build. Mater..

[56] Saravana R.M.K., Sumathi A. (2017). Effect of fly ash in fiber reinforced concrete composites. Jordan J. Civ. Eng..

[57] Challoob M.A., Srivastava V., Materials A. (2013). Effect of Fly Ash and Steel Fibre on Portland Pozzolana Cement Concrete. Int. J. Eng. Trends Technol..

[58] Saha A.K. (2018). Effect of class F fly ash on the durability properties of concrete. Sustain. Environ. Res..

[59] Roshani M.M., Kargar S.H., Farhangi V., Karakouzian M. (2021). Predicting the effect of fly ash on concrete’s mechanical properties by ann. Sustainability.

[60] Ahmad A., Chaiyasarn K., Farooq F., Ahmad W., Suparp S., Aslam F. (2021). Compressive strength prediction via gene expression programming (Gep) and artificial neural network (ann) for concrete containing rca. Buildings.

[61] Wang Q., Ahmad W., Ahmad A., Aslam F., Mohamed A., Vatin N.I. (2022). Application of Soft Computing Techniques to Predict the Strength of Geopolymer Composites. Polymers.

[62] Zhu A.Y., Ahmad W., Ahmad N.I., Vatin A.M., Mohamed D.F. (2022). Predicting the Splitting Tensile Strength of Recycled Aggregate Concrete Using Individual and Ensemble Machine Learning Approaches. Crystals.

[63] Ahmad A., Ahmad W., Chaiyasarn K., Ostrowski K.A., Aslam F., Zajdel P., Joyklad P. (2021). Prediction of geopolymer concrete compressive strength using novel machine learning algorithms. Polymers.

[64] Leinweber D.J. (2007). Stupid data miner tricks: Overfitting the S&P500. J. Investig..

[65] Kohavi R. (1995). A Study of Cross-Validation and Bootstrap for Accuracy Estimation and Model Selection. Int. Jt. Conf. Artif. Intell..

[66] Farooq F., Ahmed W., Akbar A., Aslam F., Alyousef R. (2021). Predictive modeling for sustainable high-performance concrete from industrial wastes: A comparison and optimization of models using ensemble learners. J. Clean. Prod..

[67] Ahmad A., Ostrowski K.A., Maślak M., Farooq F., Mehmood I., Nafees A. (2021). Comparative study of supervised machine learning algorithms for predicting the compressive strength of concrete at high temperature. Materials.

[68] Leung K.W.K., Pan Z.L., Warner D.H. (2016). Kohn-Sham density functional theory prediction of fracture in silicon carbide under mixed mode loading. Model. Simul. Mater. Sci. Eng..

[69] Leung K.W.K., Pan Z.L., Warner D.H. (2014). Atomistic-based predictions of crack tip behavior in silicon carbide across a range of temperatures and strain rates. Acta Mater..

[70] Saroukhani S., Nguyen L.D., Leung K.W.K., Singh C.V., Warner D.H. (2016). Harnessing atomistic simulations to predict the rate at which dislocations overcome obstacles. J. Mech. Phys. Solids.

[71] Kiani S., Leung K.W.K., Radmilovic V., Minor A.M., Yang J.M., Warner D.H., Kodambaka S. (2014). Dislocation glide-controlled room-temperature plasticity in 6H-SiC single crystals. Acta Mater..

[72] Ilawe N.V., Zimmerman J.A., Wong B.M. (2015). Breaking badly: DFT-D2 gives sizeable errors for tensile strengths in palladium-hydride solids. J. Chem. Theory Comput..

