# Supplementary material for: Compressive Strength of Steel Fiber-Reinforced Concrete Employing Supervised Machine Learning Techniques
Source: Materials (Basel). 2022 Jun 14;15(12):4209. doi: 10.3390/ma15124209 (PMC9228203; doi:10.3390/ma15124209)
Supplement: Supplementary file 1 [file materials-15-04209-s001.zip › materials-1734507-supplementary.pdf]

materials MDPI

Supplementary Materials

Compressive Strength of Steel Fiber–Reinforced Concrete Employing Supervised Machine Learning Techniques

Yongjian Li [1], Qizhi Zhang [2,*], Paweł Kamiński [3,*], Ahmed Farouk Deifalla [4], Muhammad Sufian [5,*], Artur Dyczko [6], Nabil Ben Kahla [7,8] and Miniar Atig [8,9]

1 James Watt Engineering School, University of Glasgow, Scotland, UK; yongjian4896@outlook.com
2 School of Architectural Engineering, Huanghuai University, Zhumadian 463000, China
3 Faculty of Civil Engineering and Resource Management, AGH University of Science and Technology, Mickiewicza 30, 30-059 Kraków, Poland;
4 Structural Engineering and Construction Management Department, Faculty of Engineering and Technology, Future University in Egypt, Cairo 11835, Egypt; ahmed.deifalla@fue.edu.eg
5 School of Civil Engineering, Southeast University, Nanjing 210096, China
6 Mineral and Energy Economy Research Institute of the Polish Academy of Sciences, J. Wybickiego 7a, 31-261 Kraków, Poland; arturdyczko@min-pan.krakow.pl
7 Department of Civil Engineering, College of Engineering, King Khalid University, Abha 61421, Saudi Arabia; nbohlal@kku.edu.sa
8 Laboratory of Systems and Applied Mechanics, Tunisia Polytechnic School, University of Carthage, La Marsa, Tunis 2078, Tunisia; miniar.atig@gmail.com
9 Department of Civil Engineering, The Higher National Engineering School of Tunis, University of Tunis, Tunis, Tunisia
* Correspondence: zdjs_zqz@163.com (Q.Z.); pkamin@agh.edu.pl (P.K.); drsufian@seu.edu.cn (M.S.)

Table S1. Dataset used for prediction.

Cement (kg/m³)	Water (kg/m³)	Sand (kg/m³)	Coarse Aggregate (kg/m³)	Superplasticizer (%)	Silica Fume (%)	Fly Ash (%)	Vf of the Hooked Steel Fiber (%)	Fiber Length (mm)	Fiber Dia (mm)	Compressive Strength MPa (28 days)	References
**440**	220	1225	366	3	0	0	0	0	0	46.6	1
**440**	220	1215	363	3.2	0	0	0.5	31	0.75	50.4	
**440**	220	1205	360	3.7	0	0	1	31	0.75	43.9	
**440**	220	1193	356	4	0	0	1.5	31	0.75	50.2	
**360**	180	738	1108	0.5	0	0	0	30	0.5	49.21	2
**360**	180	738	1108	0.5	0	0	0.5	30	0.5	39.14	
**360**	180	738	1108	0.5	0	0	1.5	30	0.5	40.77	
**360**	180	738	1108	0.5	0	0	2	30	0.5	40.61	
**680**	180	582	872	1.5	15	0	0	30	0.5	90.14	
**680**	180	582	872	1.5	15	0	0.5	30	0.5	90.76	
**680**	180	582	872	1.5	15	0	1.5	30	0.5	95.01	
**680**	180	582	872	1.5	15	0	2	30	0.5	96.54	
**400**	242	663	1065	0	40	0	0.25	60	0.9	26.1	3
**400**	242	663	1065	0	40	0	0.375	60	0.9	26.2	
**400**	242	663	1065	0	40	0	0.5	60	0.9	27	
**400**	198	663	1065	0	40	0	0.25	60	0.9	34.6	
**400**	198	663	1065	0	40	0	0.375	60	0.9	37.4	
**400**	198	663	1065	0	40	0	0.5	60	0.9	37.3	
**400**	165	663	1065	4.4	40	0	0.25	60	0.9	46.7	
**400**	165	663	1065	4.4	40	0	0.375	60	0.9	48.7	
**400**	165	663	1065	4.4	40	0	0.5	60	0.9	48.2	
**400**	152	835	1047	0	0	0	0	0	0	32.4	4
**400**	152	835	1047	0	0	0	0.5	40	0.615	33.4	
**400**	152	835	1047	0	0	0	1	40	0.615	37.3	
**400**	152	835	1047	0	0	0	0.5	60	0.75	34.1	
**400**	152	835	1047	0	0	0	1	60	0.75	38.5	
**400**	152	835	1047	0	5	0	0	0	0	36.4	
**400**	152	835	1047	0	5	0	0.5	40	0.615	38.3	
**400**	152	835	1047	0	5	0	1	40	0.615	48.1	
**400**	152	835	1047	0	5	0	0.5	60	0.75	41.4	
**400**	152	835	1047	0	5	0	1	60	0.75	45.7	
**400**	152	835	1047	0	10	0	0	0	0	56.2	
**400**	152	835	1047	0	10	0	0.5	40	0.615	60.4	
**400**	152	835	1047	0	10	0	1	40	0.615	66.9	
**400**	152	835	1047	0	10	0	0.5	60	0.75	59.7	
**400**	152	835	1047	0	10	0	1	60	0.75	63.7	
**400**	152	835	1047	0	15	0	0	0	0	60.1	
**400**	152	835	1047	0	15	0	0.5	40	0.615	66.5	
**400**	152	835	1047	0	15	0	1	40	0.615	69.3	
**400**	152	835	1047	0	15	0	0.5	60	0.75	63.2	
**400**	152	835	1047	0	15	0	1	60	0.75	70.5	
**520**	172	725	853	0	10	0	0	0	0	99.2	5
**520**	172	725	853	0	10	0	0	0	0	99.2	
**512**	169	714	841	0	10	0	1.5	60	0.75	98.7	
**510**	168	710	836	0	10	0	2	30	0.5	98.9	
**510**	168	710	836	0	10	0	2	60	0.75	95.3	
**550**	137.5	682	1050.5	0.25	0	0	0	40	0.62	85.5	6
**550**	137.5	682	1050.5	0.25	0	0	0.5	40	0.62	87.8	
**550**	137.5	682	1050.5	0.25	0	0	1	40	0.62	90.3	
**550**	137.5	682	1050.5	0.25	0	0	1.5	40	0.62	92.7	
**550**	137.5	682	1050.5	0.25	0	0	0.5	50	0.62	87	
**550**	137.5	682	1050.5	0.25	0	0	1	50	0.62	90.7	
**550**	137.5	682	1050.5	0.25	0	0	1.5	50	0.62	93	
**550**	137.5	682	1050.5	0.25	0	0	0.5	60	0.75	86.6	
**550**	137.5	682	1050.5	0.25	0	0	1	60	0.75	91.6	
**550**	137.5	682	1050.5	0.25	0	0	1.5	60	0.75	93.8	
**450**	157.5	715.5	1053	0.15	0	0	0	40	0.62	61.7	

**450**	157.5	715.5	1053	0.15	0	0	0.5	40	0.62	69.4	
**450**	157.5	715.5	1053	0.15	0	0	1	40	0.62	72.3	
**450**	157.5	715.5	1053	0.15	0	0	1.5	40	0.62	75.9	
**450**	157.5	715.5	1053	0.15	0	0	0.5	50	0.62	73.8	
**450**	157.5	715.5	1053	0.15	0	0	1	50	0.62	76.4	
**450**	157.5	715.5	1053	0.15	0	0	1.5	50	0.62	77.3	
**450**	157.5	715.5	1053	0.15	0	0	0.5	60	0.75	68	
**450**	157.5	715.5	1053	0.15	0	0	1	60	0.75	70.6	
**450**	157.5	715.5	1053	0.15	0	0	1.5	60	0.75	78.6	
**350**	157.5	798	1078	0.12	0	0	0	40	0.62	52.4	
**350**	157.5	798	1078	0.12	0	0	0.5	40	0.62	60.7	
**350**	157.5	798	1078	0.12	0	0	1	40	0.62	64.1	
**350**	157.5	798	1078	0.12	0	0	1.5	40	0.62	62.3	
**350**	157.5	798	1078	0.12	0	0	0.5	50	0.62	55.8	
**350**	157.5	798	1078	0.12	0	0	1	50	0.62	62.3	
**350**	157.5	798	1078	0.12	0	0	1.5	50	0.62	64.2	
**350**	157.5	798	1078	0.12	0	0	0.5	60	0.75	58.9	
**350**	157.5	798	1078	0.12	0	0	1	60	0.75	59.7	
**350**	157.5	798	1078	0.12	0	0	1.5	60	0.75	66.2	
**360**	180	738	1108	0.5	0	0	0	30	0.5	49.21	
**360**	180	738	1108	0.5	0	0	0.5	30	0.5	39.14	
**360**	180	738	1108	0.5	0	0	1	30	0.5	40.77	
**360**	180	738	1108	0.5	0	0	2	30	0.5	40.61	
**680**	180	582	872	1.5	15	0	0	30	0.5	90.14	7
**680**	180	582	872	1.5	15	0	0.5	30	0.5	90.76	
**680**	180	582	872	1.5	15	0	1	30	0.5	95.01	
**680**	180	582	872	1.5	15	0	2	30	0.5	96.54	
**323**	184	788	993	0	0	0	0	60	0.9	32.6	
**323**	184	788	993	0	0	0	1	60	0.9	33.7	
**323**	184	788	993	0	0	0	1.5	60	0.9	34.1	
**323**	184	788	993	0	0	0	2	60	0.9	37.4	
**323**	184	788	993	0	0	0	1	60	0.8	32.7	
**323**	184	788	993	0	0	0	1.5	60	0.8	36	8
**323**	184	788	993	0	0	0	2	60	0.8	33.5	
**323**	184	788	993	0	0	0	1	60	0.7	33.7	
**323**	184	788	993	0	0	0	1.5	60	0.7	36.9	
**323**	184	788	993	0	0	0	2	60	0.7	32.2	
**400**	220	1020	560	0	0	0	1	30.5	0.49	29.1	
**400**	220	1020	560	0	0	0	1.5	30.5	0.49	29.1	
**400**	220	1020	560	0	0	0	2	30.5	0.49	29.1	
**400**	220	1020	560	0	0	0	1	50.97	0.7	29.1	
**400**	220	1020	560	0	0	0	1.5	50.97	0.7	29.1	9
**400**	220	1020	560	0	0	0	2	50.97	0.7	29.1	
**468**	220	1008	449	0	0	0	1	30.5	0.49	51	
**468**	220	1008	449	0	0	0	1.5	30.5	0.49	51	
**468**	220	1008	449	0	0	0	2	30.5	0.49	51	
**430**	133	739	1052	9	43	0	0	35	0.55	85	
**430**	133	739	1052	9	43	0	0.5	35	0.55	91	
**430**	133	739	1052	9	43	0	1	35	0.55	95	10
**430**	133	739	1052	9	43	0	1.5	35	0.55	98	
**430**	133	739	1052	9	43	0	2	35	0.55	96	
**666**	200	900	540	0	0	0	0.5	30	0.5	58.2	
**666**	200	900	540	0	0	0	1	30	0.5	68.6	
**666**	200	900	540	0	0	0	1.5	30	0.5	63.6	
**666**	200	900	540	0	0	0	2	30	0.5	68.2	
**666**	200	900	540	0	0	0	0.5	30	0.5	64.5	
**666**	200	900	540	0	0	0	1	30	0.5	63.2	11
**666**	200	900	540	0	0	0	1.5	30	0.5	64.3	
**666**	200	900	540	0	0	0	2	30	0.5	65.4	
**666**	200	900	540	0	0	0	0.5	30	0.5	66.6	
**666**	200	900	540	0	0	0	1	30	0.5	67.1	

**666**	200	900	540	0	0	0	1.5	30	0.5	67.6	
**666**	200	900	540	0	0	0	2	30	0.5	65.5	
**370**	170	673	1150	0	0	0	0	60	0.75	45.3	12
**370**	170	673	1150	0	0	0	0.5	60	0.75	47.7	
**370**	170	673	1150	0	0	0	0.76	60	0.75	52.1	
**370**	170	673	1150	0	0	0	1	60	0.75	52	
**500**	142	674	1100	2.5	0	0	0	60	0.75	72	
**500**	142	674	1100	2.5	0	0	0.5	60	0.75	76.3	
**500**	142	674	1100	2.5	0	0	0.76	60	0.75	76.2	
**500**	142	674	1100	2.5	0	0	1	60	0.75	77.9	
**540**	270	1350	0	0.4	0	0	0	13	0.2	27.62	13
**540**	270	1350	0	0.4	0	0	0.4	13	0.2	28.67	
**540**	270	1350	0	0.4	0	0	0.6	13	0.2	28.47	
**540**	270	1350	0	0.4	0	0	0.8	13	0.2	29.16	
**400**	190	610	1130	0	0	0	0	30	0.54	29.8	14
**400**	190	600	1120	0	0	0	0.5	30	0.54	30.5	
**400**	190	600	1110	0	0	0	1	30	0.54	31.2	
**400**	190	590	1110	0	0	0	1.5	30	0.54	32.3	
**450**	158	610	1140	2.85	0	0	0	30	0.54	56	
**450**	158	610	1130	2.85	0	0	0.5	30	0.54	57	
**450**	158	600	1120	2.85	0	0	1	30	0.54	57.8	
**450**	158	600	1110	2.85	0	0	1.5	30	0.54	59.4	
**450**	140	630	1170	3.22	11.12	0	0	30	0.54	72.4	
**450**	140	625	1160	3.22	11.12	0	0.5	30	0.54	73.6	
**450**	140	620	1150	3.22	11.12	0	1	30	0.54	74.8	
**450**	140	615	1140	3.22	11.12	0	1.5	30	0.54	77	
**372**	160	750	1140	2.2	7.5	0	0	30	0.5	56.1	15
**372**	160	750	1140	2.2	7.5	0	0.5	30	0.5	59.2	
**520**	156	860	886	1	0	0	0	0	0	82.6	16
**468**	156	847	873	1.1	10	0	0.25	60	0.75	92.3	
**468**	156	844	870	1.1	10	0	0.5	60	0.75	93.8	
**468**	156	841	867	1.2	10	0	0.75	60	0.75	95	
**468**	156	838	863	1.2	10	0	1	60	0.75	98.7	
**400**	140	756	1135	1	0	0	0	35	0.55	77.1	17
**400**	140	754	1131	1	0	0	0.25	35	0.55	79.4	
**400**	140	751	1127	1	0	0	0.5	35	0.55	78.2	
**400**	140	746	1119	1	0	0	1	35	0.55	80.5	
**400**	140	740	1111	1	0	0	1.5	35	0.55	81	
**340**	140	750	1124	1	0	15	0	35	0.55	67.8	
**340**	140	747	1120	1	0	15	0.25	35	0.55	69.4	
**340**	140	744	1116	1	0	15	0.5	35	0.55	68.3	
**340**	140	739	1108	1	0	15	1	35	0.55	71.7	
**340**	140	734	1100	1	0	15	1.5	35	0.55	72.7	
**280**	140	742	1114	1	0	30	0	35	0.55	63.6	
**280**	140	740	1109	1	0	30	0.25	35	0.55	61.8	
**280**	140	737	1106	1	0	30	0.5	35	0.55	64.4	
**280**	140	732	1097	1	0	30	1	35	0.55	65	
**280**	140	726	1090	1	0	30	1.5	35	0.55	60.7	

References

1. Soulioti, D.V.; Barkoula, N.M.; Paipetis, A.; Matikas, T.E. Effects of fibre geometry and volume fraction on the flexural behaviour of steel-fibre reinforced concrete. *Strain* **2011**, *47*, e535–e541.
2. Yoo, D.Y.; Yoon, Y.S.; Banthia, N. Flexural response of steel-fiber-reinforced concrete beams: Effects of strength, fiber content, and strain-rate. *Cem. Concr. Compos.* **2015**, *64*, 84–92.
3. Lee, J.H.; Cho, B.; Choi, E. Flexural capacity of fiber reinforced concrete with a consideration of concrete strength and fiber content. *Constr. Build. Mater.* **2017**, *138*, 222–231.
4. Köksal, F.; Altun, F.; Yiğit, I.; Sahin, Y. Combined effect of silica fume and steel fiber on the mechanical properties of high strength concretes. *Constr. Build. Mater.* **2008**, *22*, 1874–1880.
5. Yoon, E.S.; Park, S.B. An experimental study on the mechanical properties and long-term deformations of high-strength steel fiber reinforced concrete. *J. Korean Soc. Civ. Eng.* **2006**, *26*, 401–409.

6. Abbass, W.; Khan, M.I.; Mourad, S. Evaluation of mechanical properties of steel fiber reinforced concrete with different strengths of concrete. *Constr. Build. Mater.* **2018**, *168*, 556–569.
7. Yoo, D.Y.; Yoon, Y.S.; Banthia, N. Predicting the post-cracking behavior of normal-and high-strength steel-fiber-reinforced concrete beams. *Constr. Build. Mater.* **2015**, *93*, 477–485.
8. Lee, H.H.; Lee, H.J. Characteristic strength and deformation of SFRC considering steel fiber factor and volume fraction. Journal of the Korea Concrete Institute **2014**, *16*, 759–766.
9. Oh, Y.H. Evaluation of flexural strength for normal and high strength concrete with hooked steel fibers. Journal of the Korea Concrete Institute **2008**, *20*, 531–539.
10. Song, P.S.; Hwang, S. Mechanical properties of high-strength steel fiber reinforced concrete. *Constr. Build. Mater.* **2004**, *18*, 669–673.
11. Jang, S.J.; Yun, H.D. Combined effects of steel fiber and coarse aggregate size on the compressive and flexural toughness of high-strength concrete. *Compos. Struct.* **2018**, *185*, 203–211.
12. Aldossari, K.M.; Elsaigh, W.A.; Shannag, M.J. Effect of steel fibers on flexural behavior of normal and high strength concrete. *Int. J. Civ. Environ. Eng.* **2014**, *8*, 22–26.
13. Dinh, N.H.; Park, S.-H.; Choi, K.K. Effect of dispersed micro-fibers on tensile behavior of uncoated carbon textile-reinforced cementitious mortar after high-temperature exposure. *Cem. Concr. Compos.* **2021**, *118*, 103949.
14. Thomas, J.; Ramaswamy, A. Mechanical properties of steel fiber-reinforced concrete. *J. Mater. Civ. Eng.* **2007**, *19*, 385–392.
15. Sivakumar, A.; Santhanam, M. Mechanical properties of high strength concrete reinforced with metallic and non-metallic fibres. *Cem. Concr. Compos.* **2007**, *29*, 603–608.
16. Afroughsabet, V.; Ozbakkaloglu, T. Mechanical and durability properties of high-strength concrete containing steel and polypropylene fibers. *Constr. Build. Mater.* **2015**, *94*, 73–82.
17. Atis, C.D.; Karahan, O. Properties of steel fiber reinforced fly ash concrete. *Constr. Build. Mater.* **2009**, *23*, 392–399.
